# Narratives of quitting and quests for teaching reform: an affective event perspective on Chinese doctoral students' intentions to drop out

**DOI:** 10.3389/fpsyg.2026.1742541

**Published:** 2026-03-13

**Authors:** Xin Li, Sanyou Shu

**Affiliations:** College of Communication Science and Art, Chengdu University of Technology, Chengdu, China

**Keywords:** affective event theory, doctoral education, dropout, higher education, teaching reform

## Abstract

In the field of higher education, the phenomenon of doctoral students dropping out has gained attention from researchers. Yet the reasons driving this phenomenon remain underexplored in the Chinese context. Guided by the Affective Event Theory, this study investigated the environmental characteristics of education that trigger the dropout emotions among doctoral students. Based on thematic analyses of the dropout narratives within a Chinese doctoral students' online community, this study reveals that certain negative yet stable environment characteristics trigger a series of negative events, resulting in doctoral students' dropout sentiment. Specifically, these characteristics include completion difficulty, a lack of academic autonomy, competition and conflict within the *shi-men* (a supervisor-centric learning community for postgraduate students), and low employment expectations. Based on the findings, this study provides suggestions to improve doctoral students' learning environment, for example, humanizing the power of supervisors and their advising process, helping doctoral students to form communities of learning that give them more channels to communicate their progress and receive feedback and support. This paper contributes to the higher education reform by offering culture-specific directions for research and by providing guidelines for the training of university leaders and supervisors.

Since China's reform and opening-up policy, postgraduate education has been viewed as a crucial means of cultivating high-level talent and enhancing national competitiveness. China's Ministry of Education (2020) clearly stated this point in the Opinions on Reforming and Developing Postgraduate Education in the New Era. Currently, China has become a large country in terms of doctoral education—the number of doctoral students in China has reached 612,500 (Ministry of Education, 2024). However, amid the continued expansion of doctoral education, the study experience of Chinese doctoral students has not improved accordingly.

Surveys indicate that the proportion of doctoral students choosing to drop out is increasing annually in China (Cheng and Qi, 2023). Dropout not only resulted in the advisors' delays in research progress and a waste of educational resources but also affected doctoral students' lives, career, and mental health, and dropout has become a common phenomenon in doctoral education worldwide (Feizi et al., 2024; Schmidt and Hansson, 2018; Wollast et al., 2023). Within the highly competitive Chinese education system, it is hard to get admission to doctoral programs. Why do these doctoral students, who are admired by many others, want to quit? The study experience of doctoral students is an essential factor that influences their decision. Their narratives can help understand their concerns.

In this context, analyzing the affective events behind Chinese doctoral students' dropout intention helps understand the needs of this group and may provide insights into improving the doctoral education system. Based on the analysis of dropout postings in an online community of Chinese doctoral students, this study proposes the following research question: what events motivate doctoral students to drop out? Drawing on the Affective Event Theory from the field of organizational management, we attempt to construct a framework for understanding the dropout phenomenon by viewing doctoral students' academic activities as a type of organizational activity. The main reasons for choosing this theory include: doctoral students' academic activities are essentially intellectual labor; the doctoral student–advisor relationship relates to the advisor's leadership style, a topic in organizational studies; and doctoral students' learning process can be viewed as organizational participation behaviors.

Guided by AET theory, this study examines Chinese doctoral students' narratives of affective events that trigger quitting, intending to identify characteristics of the Chinese academic environment that generate negative events and emotions and ultimately lead to dropout intentions. As a basic paradigm of human communication, narratives reflect how individuals interpret and reflect on their life experiences, giving logical meaning to the individual's thoughts and behaviors (Fisher, 1985). The study deepens understanding of the relationships among environmental characteristics, incidents, and emotions. Based on the findings, we offer suggestions for improving doctoral education in China. The study first reviews the relevant literature on factors influencing doctoral student dropout, Chinese doctoral students' academic activities, and the affective event theory, and then describes the process of data collection and analysis. It subsequently reports the findings and discusses the theoretical and practical contributions of the study.

## Literature review

### Factors influencing doctoral student dropout

The dropout of PhD students is a major concern in higher education. Dropout incidents are thought to have occurred due to doctoral students' personal factors and external environmental factors (Sverdlik et al., 2018). Personal factors include perceived competence and perceived pressure. Research has found that the greater the progress doctoral students make in their courses and the more papers they publish in academic journals and present at research conferences, the less likely they are to consider dropping out (Litalien and Guay, 2015). Academic engagement strengthens doctoral students' perceptions of personal resources, increases their sense of wellbeing, thereby enhancing satisfaction with their doctoral study (Cao et al., 2024; Corcelles-Seuba et al., 2023). On the other hand, the greater the academic pressure doctoral students perceive, the more likely they are to experience self-doubt and dropout (Feizi et al., 2024).

The external environment also significantly influences doctoral students' dropout. One area of focus is the relationship between doctoral students and their supervisors. As individuals who provide academic advice during the student's learning phase, the supervisor plays a crucial role in the student's academic integration. Studies have found that the degree of “matching” between doctoral students and their supervisors, including the degree of rapport in interpersonal relationships and academic matching (Travaglianti et al., 2018; Van Rooij et al., 2021), supervisors' support (Corcelles-Seuba et al., 2023; Feizi et al., 2024; Gruzdev et al., 2020; Kis et al., 2022), and supervisors' fairness/recognition (Travaglianti et al., 2018) are significantly related to doctoral students' dropout intentions. If the academic environment of the department does not match the expectations of doctoral students, it can hinder their progress in research and learning (Cai et al., 2024; Leijen et al., 2016). Studies have found that those who experience less healthy research environments (e.g., peer bullying, unethical supervision) are more likely to consider quit (Kis et al., 2022; Peng et al., 2022).

The extent of these factors' influence varies by discipline and societal context. For example, humanities students are more concerned with emotional support (Glorieux et al., 2025), while medical students exhibit a stronger intent to drop out in China (Peng et al., 2022). International doctoral students face the added challenge of adapting to new academic environment and culture. Laufer and Gorup (2019) found that international doctoral students are more likely to experience dropout emotions when being regarded as “foreigners” and “academic others.”

### Chinese doctoral students' academic activities

Current Chinese doctoral education is similar to that of Western countries, particularly the United States. The specific requirements for graduation are formulated by the degree-granting institutions, guided by the Degree Management Regulations of the Ministry of Education. Generally, doctoral students need to: (a) pass coursework assessments and obtain sufficient credits; (b) publish a certain number of articles; and (c) complete a doctoral dissertation, pass the blind review organized by the Ministry of Education, and pass the defense. Therefore, doctoral students need to constantly maintain communication with their advisors and the graduate program to get update about their progress.

In addition to the formal organizing, it is also important for doctoral students to know about the informal organizing centered around the supervisor, called *shi-men* (师门, an academic group consisting of the supervisor and all of his or her graduate students). Chinese doctoral education mainly implements a supervisor-centered system. Under this system, the supervisor-centered *shi-men* has an important impact on doctoral students. *Shi-men* is the main place for graduate students and the supervisor to engage in academic discussions and other exchanges, with a much higher frequency of interaction and degree of social support than formal organizing (Lin and Chao, 2019). Research has confirmed the positive functions of *shi-men*, such as helping “newcomers” build interpersonal relationships and develop a sense of belonging (Guo et al., 2020); providing seminar opportunities (Gu and Pu, 2023); and helping doctoral students acquire disciplinary knowledge, academic norms, and research methods (Gu and Pu, 2023; Lin and Chao, 2019).

Although *shi-men* does not differ much from Western scholars' teams centered around the supervisor, due to the influence of Confucianism, *shi-men* has formed unique organizational characteristics. A very high degree of respect for the supervisor is a basic requirement in the supervisor-student relationships and is considered the primary virtue of students (Guo et al., 2020; Tan, 2015). Secondly, the demand for research achievements is likely to result in relationship alienation among the students. Publishing a certain number of articles in quality journals is one of the graduation requirements for Chinese doctoral students, and the research achievements are linked to material interests, such as scholarships, graduation, and employment. Most doctoral students rely on the supervisor's advice and allocation of research resources. Because limited resources are difficult to distribute equally, members' different positions in the *shi-men* affect their emotions for academic work (Lin and Chao, 2019). Table 1 summarizes the major differences between Chinese and Western/U.S. doctoral education.

**Table 1:** Comparison of Chinese and Western/U.S. doctoral Education.

**Requirement item**	**Chinese doctoral education**	**Western/U.S. doctoral education**
Journal article publication	Yes/Mandatory	No/Voluntary
External review for doctoral dissertation	Yes/Mandatory	No/Not required
Autonomy in research	No/Determined by Supervisor	Yes
Supervisor-student relationship	Complicated/*Shi-men*	Simple

With the younger generation's demystification of doctoral study, the labor nature of academic activities resonates among many doctoral students. Many believe that, in addition to being learners and researchers, they are more of “academic laborers” serving the supervisor. Awareness of the mixed identity, i.e., a combination of academic identity and basic labor, has become a self-deprecation for many doctoral students. The negotiation between multiple identities has become a key factor affecting the psychological health of doctoral students (Sun and Li, 2023).

### Theoretical framework: affective event theory

This study uses the Affective Event Theory (AET) as a guide to explore doctoral students' organizational activities. Developed by Weiss and Cropanzano (1996), this theory explains the effect of exogenous factors on individuals' emotions and feelings in the workplace. This theory suggests that incidents triggered by the characteristics of job environment affect individuals' emotions, which affect their work behaviors (e.g., job performance) and attitudes (e.g., job satisfaction) (Ghasemy et al., 2021). An individual's emotional response is determined by their cognitive assessment of the incident that has occurred, including assessing (a) the relevance of the incident to their wellbeing, (b) the importance of the incident, and (c) the extent to which the incident benefits them. For example, when an incident is considered relevant and important, and assessed as beneficial, the individual will experience positive emotional states; conversely, relevant, important, but detrimental incidents will trigger negative emotional states. Studies have confirmed the causal effect between work incidents and emotions (Ghasemy et al., 2021; Schulz, 2013), and have found that individuals' emotional states in the workplace predict their work attitudes and behaviors, such as the intention to resign (Ashkanasy et al., 2014; Christensen et al., 2023). Researchers assessed a wide range of emotional incidents, for example, arousal or stress incidents (Lee et al., 2021), positive and negative interpersonal interactions (Christensen et al., 2023; Glasø et al., 2010). The characteristics of the work organization, including job autonomy, promotion opportunities, benefits, and leadership styles, are considered important aspects triggering work incidents (Luo and Chea, 2018).

From the perspective of AET, there are similarities between ordinary work and doctoral student learning. For example, collaborating on research with the supervisor and peers in the *shi-men* and the supervisor's style are similar to the peer relationships and leadership styles involved in the workplace. Some doctoral students face interpersonal conflicts and competition with their peers in the *shi-men* and are dissatisfied with the supervisor's task allocation, which may trigger negative emotions among them (Sverdlik et al., 2018). In addition, doctoral students' reactions to certain characteristics of the university may also be similar to employees' reactions to the characteristics of the work organization. Pressure from doctoral study (e.g., publication) and low employment expectations are similar to work pressure and fewer promotion opportunities in the workplace.

This study employs theme analysis to explore doctoral students' dropout intentions. According to AET theory, dropout intention, as a manifestation of negative emotions, is caused by specific negative incidents triggered by environmental characteristics, interpersonal relationships, and leadership styles. Therefore, this study focuses on the explanations of specific incidents by doctoral students that led to their dropout intentions.

## Method

Narratives in online communities provide research data in a naturalistic form, which is the key to reflecting individuals' true thoughts (Li and Xu, 2023, 2024, 2025; Xu and Li, 2023). Therefore, this study chose the Chinese doctoral students' online community “Doctoral Student, I'm Dropping Out” as the research object. Created in March 2019 on one of China's largest social media platforms, “Douban,” the community has attracted more than 35,000 members to date, making it the largest doctoral students' community in Douban. Consistent with the name, this community aims to provide “all doctoral students who want to drop out, are dropping out, and have dropped out” with “those dark, unbearable experiences and stories.” The community members have generated rich narratives surrounding the idea of dropping out, which become the data source for this study.

This study used Python to crawl the data in the group. In order to ensure the quality of the content and eliminate advertisement posts, the study selected posts with replies, obtaining 1,547 data items in total. As this study aims to explore the driving incidents that led to dropout ideas among doctoral students, the authors screened and selected the data according to the following criteria: (1) the poster must declare their doctoral student status and clearly express the intention of “dropping out,” or have put this idea into action; (2) the post must provide a detailed description of the events triggering the dropout idea; and (3) the post content must have sufficient information about the dropout. After screening, a total of 506 valid data items were obtained.

The Affective Event Theory was used as a framework to guide the analysis of the affective events that led to doctoral students' intention to drop out. The analysis focused on identifying the themes in the narratives that relate to the narrators' dropout intention. MAXQDA was used for data coding. Analysis of narratives has many paths, the main one of which is theme analysis. Riessman (2008) believes that theme analysis emphasizes the themes of the narratives. Similar to the grounded theory approach, data collection and analysis were performed simultaneously. The authors carried out three rounds of coding together, including open coding, focused coding, and theoretical theme coding. First, during the open coding process, the authors remained open to all possible theoretical directions. Second, during the focused coding stage, based on the similarity and repetition patterns between codes, similar codes were grouped to form categories and further combined into sub-themes. Finally, during the thematic coding process, themes and their sub-themes were created by noting the relationships between the themes. The authors find that four overarching themes can govern all the codes and data: completion difficulty, a lack of academic autonomy, a competitive *shi-men* environment, and low employment expectations.

The analysis used the original Chinese text collected from the Chinese online community, and the excerpts used in the article were translated into English. This study was exempt from Institutional Review Board approval because the data used were only from a publicly available online community. The authors also changed the pseudonyms of the online community members to further protect their privacy. Therefore, this study has complied with ethical requirements.

## Findings

AET regards work autonomy, leadership style, promotion opportunities and benefits as the main factors that trigger work incidents and lead to changes in employees' emotions. This study found that doctoral students' experiences at Chinese universities are similar to those of employees in work organizations. Negative emotions and dropout intentions among doctoral students are primarily caused by a negative learning environment characterized by completion difficulty, a lack of academic autonomy, a competitive *shi-men* environment, and low employment expectations. Table 2 shows the above themes, their sub-themes, and the frequency they appear in the posts.

**Table 2:** Narrative themes and sub-themes.

**Narrative theme**	**Sub-theme**	**The number of posts showing the sub-theme**
Completion difficulty	Difficulty in publishing	108
	Slow progress in dissertation	64
Lack of academic autonomy	Authoratative/Coercive supervising style	97
	Lack of appropriate advice	62
Competitive S*hi-men* environment	Peer pressure in Shi-men	21
	Conflict in Shi-men	18
Low employment expectations	Pessimistic prospects	25
	Past false expectations	19

### Completion difficulty: a harsh achievement-oriented system

As employees in the workplace consider their promotion opportunities, doctoral students also consider their graduation opportunities. To ensure the quality of doctoral education and compete for ranking, Chinese universities set demanding academic requirements for graduation. Among the requirements, the coursework assessment is relatively simple. The discussions in the community by doctoral students regarding the difficulty of graduation mainly focus on article publication and PhD dissertations.

Chinese universities usually require doctoral students to publish two or more articles in designated journals, such as the Chinese Social Sciences Citation Index (CSSCI) journals and the Social Sciences Citation Index (SSCI) journals for students in humanities and social sciences. This is related to the “metrics culture” and research evaluation in academia—universities need more publications to achieve better ranking. Although doctoral students in the U.S. and Europe also try to publish to be competitive in the job market, publishing in academic journals is not a requirement for graduation. The PhD dissertation involves multiple “inspection” stages from the proposal defense to the pre-defense of the dissertation, the blind review by outside reviewers (usually three to five professors) arranged by the Ministry of Education, and the final defense, and faces the risk of being rejected at any stage. For example, “momo,” a fifth-year doctoral student, mentioned that her dissertation proposal was rejected, which directly affected her confidence in subsequent work and led to the idea of dropping out:

The dissertation proposal was revised for more than a month, and I prepared for the defense for two months. The supervisor rejected it in 2 minutes. It was really painful. I had the idea of dropping out. I felt that I hadn't entered the field of research after doing research for so long… Many students failed. I attended a doctoral pre-defense, and the student was asked to rewrite his dissertation. (Post 76)

Development is an individual's need and a way to realize social value. As students, progress in their studies is positive feedback, helping to build confidence in their identity. The publish-or-fail graduation policy in Chinese doctoral education hinders students' learning. Some doctoral students who had no research output gradually developed low self-esteem and lost confidence in their academic abilities. For example, a doctoral student who lacks publications wrote about changes in his self-evaluation:

I have a paper from previous work, which has been submitted and rejected several times. Even if it is accepted, it cannot be accepted by good journals… I'm extremely unconfident in doing anything now. I was a very positive person before, but now I have reached the point of avoiding everything, not wanting to work, not wanting to socialize, and not even wanting to see people. I feel like a failure. (Post 49)

Because of the delay in publishing articles, many doctoral students failed to graduate on time. They believe that their lives have deviated from the normal social rhythm, falling into “stagnation.” The limited duration of study, usually 4 years with a stipend, has accelerated the panic of being left behind, and there are voices in the community worrying about the time costs. For example, “mokoko” mentioned this sense of urgency:

I was supposed to be in a four-year program, and I have already delayed graduation for a year. I found out that I have no academic ability in the second year, but at that time I was still thinking that I could continue. Until now, time has strangled me tightly. (Post 221)

### Lack of academic autonomy: the power imbalance between doctoral students and supervisors

In addition to self-learning, doctoral students' research activities rely on the guidance of the supervisor. According to the doctoral students in the community, the two opposite phenomena of over-control and absence of supervision by the supervisor are key factors affecting their perceptions of academic autonomy, and they embody the power imbalance between doctoral students and supervisors.

Supervisors have authority in doctoral student-supervisor relationship. In China, this stems not only from the influence of Confucianism (Guo et al., 2020) but also from the present educational system. China's doctoral student supervision mainly relies on a sole-supervisor responsibility system. The supervisor is responsible for the entire stages of training for doctoral students. Therefore, over-control by supervisors occurs frequently. Most doctoral students in the community mentioned that studying for a doctorate is not an equal academic dialogue with the supervisor but a one-way submission. For example, “TuaBaba,” a first-year doctoral student, shared the excessive control by the supervisor:

The supervisor does not allow (us) to have our own thinking. For example, when I discussed academic issues with him, he always said, “Your ideas are wrong, the literature you read is wrong”... Every step of the experiment must be confirmed by him before proceeding to the next step, and there is no room to exert initiative. Everyone in the research group is like an experimental machine. (Post 42)

“Ping Wujingchu,” a third-year doctoral student, also faces the same problem:

The requirements for publication have been satisfied, and I finished a 60,000-word dissertation proposal. The supervisor said there was no theoretical support. But I wrote a lot of theory stuff, and asked other professors, who all felt that there was no problem, but the supervisor still insisted on not letting me proceed with the proposal defense. (Post 111)

In the end, she missed the opportunity for the proposal defense, and the subsequent dissertation work could not be conducted. In addition, some doctoral students were assigned tasks unrelated to their studies, such as helping supervisors apply for projects, preparing course materials, and handling personal affairs, which affected the progress of their studies. The academic over-control often invades the personal life domain of doctoral students due to the lack of boundaries. Some students complain that activities during holidays are criticized by supervisors as “not engaging in proper work.” A student mentioned, “Except for the Spring Festival, there is no chance to go home during the holidays” (Post 86). Some doctoral students wrote that the supervisor's academic assessment sometimes shifted to accusations of personality, hurting their self-esteem. For example, “Mokoko” suffered from depression and anxiety due to this,

“I have a relatively poor foundation on theory. I thought that I would work hard, but the supervisor said that I was rubbish. I may have buried the seeds of depression and anxiety at that time.” (Post 221)

The opposite phenomenon to over-control is the absence of supervision. The supervisor is expected to play a leading role in the academic development of the doctoral student. However, doctoral students without appropriate guidance from the supervisor find it difficult to make progress and are unable to meet the requirements for graduation. For example, “Kite,” a sociology Ph.D. student, wrote in the community:

80% of his doctoral students dropped out, and 20% were delayed. The supervisor rarely helped us to look at the dissertation. When we met offline, he always talked about other things, only emphasizing that my research must make outstanding contributions to the discipline and social development. I sent the electronic version of the dissertation to him many times, and only received a “Pay attention to the format” reply, and when I continued to ask questions, he disappeared. If the supervisor does not provide guidance, how can we achieve results by relying on those few courses? (Post 126)

Although academic achievements are very important for doctoral students, as emotional subjects, they still long for a positive emotional connection with the supervisor in addition to academic exchanges, rather than just a pure “work” relationship. In China, “relationships” mean social resources and are indispensable elements for successfully completing tasks. This cultural concept also affects doctoral students' understanding of supervisor-student relationships. A good supervisor-student relationship, therefore, is regarded as important for the student to succeed. Conversely, the lack of a good emotional connection naturally means the loss of “opportunity.” Some doctoral students attribute the poor progress of their studies to the lack of this emotional connection:

I have an introverted personality, and I'm not very good at dealing with people, which has led to a poor relationship with my supervisor from the beginning. There has been no progress on the topic at all. Later, when I discussed a few directions with the supervisor, all of them were rejected, and no guidance or suggestions were given. (Post 79)

### Competition and conflict in *Shi-men*

Doctoral students' learning experience is affected by the *shi-men* in which they are located. Research has confirmed that the *shi-men*, as a small academic community, can improve the research capabilities of doctoral students through positive teacher-student interaction (Lechuga, 2011; Nuis et al., 2023) and peer interaction (Zheng and Wang, 2021). However, the high output pressure and limited academic resources give *shi-men* competitive characteristics—students with high research capabilities are at the core of the *shi-men* and receive a high degree of attention and incentives from the supervisor; students with mediocre academic backgrounds tend to be marginalized (He et al., 2024). Being in different positions and circles in the *shi-men* causes peer pressure. For example, “Seeker” wrote about the peer pressure he experienced:

My project has not made any substantive progress, so the advisor arranged for an excellent student to help me. The student did experiments perfectly, but my results were always unsatisfactory, which put me under a lot of pressure, and I was anxious about the comparison every day. Moreover, the advisor's arrangement made us unsure who would write a dissertation on the topic, although I have done experiments on this topic for two and a half years. It's very stressful. (Post 113)

Although Chinese PhD programs adopt a class system, this formal organization is loosely coupled. *Shi-men* is the main place for doctoral students to address their social and emotional needs. However, the competitive environment makes it difficult for the *shi-men* to form a united atmosphere, and members may be more concerned with individual interests rather than collective development. Similar to the negative events that employees experience at work, which reduce employees' perception of organizational support, unpleasant incidents that occur in the *shi-men* reduced doctoral students' perception of support. Therefore, under such an environment, an intention to withdraw is likely to arise. A doctoral student shared his story of withdrawing from the lab:

The work schedule of the lab is 997 [9 a.m. to 9 p.m., 7 days a week], and everyone spends most of the time checking their phones and reading novels at their work stations, and the relationship between each other is also very average. The 10-person team is divided into several groups, and my research has just begun. A senior fellow student thinks that our experiments conflict. Each in the lab plays their own games, and I can't make any friends. The doctoral students' circle is really small. They all have their busy experimental schedules, and it's so lonely. Several times I burst into tears and hid in the dormitory to cry, and it took a long time to adjust my emotions. (Feixu, Post 59)

Doctoral students' negative incidents with the supervisor also cause them to make negative evaluations of academia. Because a supervisor's style is difficult to change, doctoral students worry that such incidents will repeat, which leads them to consider dropout. “Jianghe,” a fourth-year doctoral student, shared a conflict with the supervisor during her doctoral study, which led to her quit:

The supervisor suddenly called me at ten o'clock in the evening and asked me about the progress of the dissertation. I truthfully said that I hadn't finished it, and he started a barrage of criticism. After scolding for a while, he told me to call my parents over to talk to him if I wanted to continue, which made me a little angry. I told him that I am an adult and there is no need to talk to my parents about anything, and he said that there was nothing more to talk about, and then he used the three threats (changing major/changing supervisor/dropping out) again. This time I also said directly that I chose to change a supervisor. My family and friends all persuaded me to bow my head to him (I had always done this in the past 3 years), but this time I felt that I was physically and mentally exhausted, and my confidence had been destroyed. Even if he agreed to let me continue, I would have no motivation to do research with him again. Looking back on the past three years, every negation and criticism has been like a straw on my back, and the recent one completely crushed me, and I can't stand up again. (Post 13)

### Negative employment expectations: a foreseeable hard career path

Like employees considering their future career, doctoral students also conduct career planning. Because universities are a relatively suitable workplace for research and teaching, doctoral students have learned about various employment opportunities in higher education, including salary and promotion requirements and opportunities and many have formed negative expectations for the future, affecting their willingness to continue with PhD studies.

The financial pressure that doctoral students feel during their study is relatively small. Chinese doctoral education is usually tuition-free, and universities provide students with a stipend. In addition, Chinese society's emphasis on education makes most families willing to provide financial support to doctoral students. However, students have to consider the income after graduation to see if it is worth spending a long time studying for a doctorate. For example, “Biscuit,” a first-year doctoral student in astronomy, gave up a high-paying job at an internet company before joining the PhD program, thinking that earning a doctorate could realize her dream, but found that the reality was not as expected, and she shared her anxiety:

I hold that good job offer (yearly income RMB 300,000+), but I chose to give it up because I passed the doctoral exam, and now I found that a Ph.D. in this major can only continue to do research at universities in the future, but university junior faculty's salaries are only RMB 100,000. I'm very anxious about whether to continue the study. (Post 28)

In addition to salary, doctoral students believe that the current employment opportunities are not optimistic. Many Chinese universities restrict the age of fresh doctoral graduates to 35 years old when recruiting, which increases their fear of the long-term doctoral study. Moreover, due to the emphasis on publish-or-perish and an increase in the number of doctoral students, universities are gradually increasing the requirements for job applicants. Some doctoral students complained that getting into an ideal university has become very difficult: “After inquiring about the entry requirements of some universities, I feel that I can't do it. Even those universities that I didn't consider before may not accept me, saying that I don't have enough publications” (Post 378). In addition, studying for a PhD allows them to know about the competitive academic environment, which makes them doubt their competence in academic work and results in the withdrawal intention. A doctoral student wrote:

Recently, I have fallen into a state of aversion to schoolwork, and now I can't see the prospect of completing the research at all. I also feel what I'm doing is very meaningless. Graduating with a doctorate and working in the hospital in the future, I will still be stuck on publishing articles. I will have endless projects to apply for, and it's already so difficult for me to complete a doctorate. (Post 112)

With the increasing pressure of publication and the competitive academic job market, universities are no longer the preferred choice for many doctoral students. Studies show that the proportion of doctoral graduates from several top universities in China who take positions in higher education is less than 50%, while the number of PhDs going to non-academic professions has increased year by year (Fan and Shen, 2024). This diversification of employment also confirms the worries of many doctoral students.

## Discussion

Drawing on the Affective Events Theory (AET), this article explores Chinese doctoral students' dropping out of the school. The study reveals that the environmental characteristics perceived by doctoral studies affected them and led to their quit by triggering a series of incidents and emotional states. Although past research has provided insights for understanding the reasons for doctoral students' dropping out (Laufer and Gorup, 2019; Peng et al., 2022; Phan, 2024), few researchers have paid attention to the students' emotions that lead to dropping out.

This study mainly makes contributions in two aspects. Firstly, guided by the AET theory, our research explored the characteristics of the Chinese academic environment that trigger negative events and emotions among doctoral students, which led to dropout intentions. Some environmental characteristics of doctoral education, such as completion difficulty, a lack of academic autonomy, competition and conflict in the *shi-men*, and lower expectations for employment opportunities, hindered doctoral students' learning experience by triggering negative events and emotions, which reduced academic satisfaction and increased their intentions to quit. Instead of discussing the impact of a specific incident on doctoral students' willingness to study, this study advocates that more attention should be paid to the role played by some stable educational environment characteristics. Similar to the ordinary work environment, doctoral students with a lack of academic autonomy and inability to develop personal abilities in academic participation tend to develop negative emotions (Cao et al., 2024). Given this, future research should focus on increasing doctoral students' autonomy and choices and enhancing their self-efficacy in the learning environment, and improving the educational system.

Secondly, our research deepens the understanding of the relationship between environmental characteristics, incidents, and emotions. The findings not only confirmed the influence of negative incidents on doctoral students' intentions to drop out, such as supervisor-student relationships (Kis et al., 2022; Travaglianti et al., 2018; Van Rooij et al., 2021) and academic stress (Feizi et al., 2024), but also found that these specific negative incidents are related to the Chinese academic environment in which they are located. The environmental characteristics and incidents mutually reinforce each other. When the incidents occur more frequently and are predictable, they become a characteristic of the learning environment. Environmental characteristics, in turn, make certain negative incidents last longer and occur more frequently.

The results of this study also have some practical implications. Doctoral education may move from traditional supervisor-focused learning to organizational participation and improve students' learning process through the optimization of the organizational environment. Based on some of the problems identified in this study, we offer the following three suggestions to improve doctoral education in China. First, humanize the power of supervisors and their advising process. The absolute power of supervisors in Chinese society, where hierarchy is highlighted, puts doctoral students in the position of “being decided.” Universities can consider setting up an academic supervisory body to regulate the supervisors' advising process, and at the same time provide a channel for doctoral students to give feedback and collect their evaluation of the supervisory effect. Although supervisors have professional responsibilities for doctoral students, universities should offer a certain number of academic courses to provide doctoral students with foundational support, to reduce their over-dependence on supervisors. As a key factor in the student-supervisor relationship and atmosphere of *shi-men*, the supervisor has the power to make many decisions regarding the student's study. The university needs to emphasize the cultivation of supervisory ability by strengthening training. Secondly, weakening or abolishing the requirement of publications, changing the training of doctoral students from “result-based evaluation” to “process-oriented evaluation.” Colleges and universities may abolish the mandatory requirement of journal article publication and focus more on the process assessment of doctoral training. Eliminating the requirements can not only reduce the pressure on students for graduation, but also change the utilitarian nature of student-supervisor and peer relationships. Recently, several Chinese universities (e.g., Tsinghua University and Peking University) have canceled the mandatory publication requirement for doctoral students to move from results-based to process-oriented education. Thirdly, attention should be paid to the mental health of doctoral students. Compared with other countries, Chinese doctoral students are characterized by low academic support and high psychological risk (Jin and Yang, 2021). Given this situation, Chinese universities may help doctoral students to form communities of learning that give them more channels to communicate their progress and receive feedback and support. Previous research also demonstrates that opportunities for PhD students to communicate their advances, receive expert feedback and interact with other researchers have a positive influence on their doctoral journey (Corcelles et al., 2019). Additionally, universities can regularly publicize general knowledge of mental health and encourage doctoral students to seek help.

## Conclusion

Guided by the Affective Event Theory, this study explored Chinese doctoral studentsh explanations of their intentions to drop out by analyzing their stories posted in an online community in China. Four types of factors were viewed to have triggered their dropout emotions: completion difficulty, a lack of academic autonomy, competition and conflict within the *shi-men*, and low employment expectations. The study deepens the understanding of the relationships among environmental characteristics, incidents, and emotions in educational contexts. The findings also have implications for public policy on support for doctoral students.

Despite the important contributions, this study also has several limitations. First, the samples come from narratives in the online communities of doctoral students. As an analysis of static texts, it is mainly based on the experiences and feelings of the members at the time of posting; therefore, it is difficult to keep track of the development of these doctoral students' dropout ideas. A netnographic study of this online community may help to resolve this issue in future research. Second, although the posts collected from the online community fit the research objectives, the study only focused on the doctoral students who have joined the online community and are willing to share their stories, while the stories of other doctoral students who have not participated in such online health communities are excluded. Thus, our research findings may not be able to reflect their interpretations of dropout. Future research can add in-depth interviews with doctoral students who have dropped out or are considering dropping out but did not voice their concerns online to further investigate the issue and provide more concrete solutions. Nevertheless, the sample in this study still has a high degree of representativeness in the doctoral student population (e.g., involving multiple universities and majors). Finally, this study did not use quantitative methods to explore further the specific causal relationships between environmental characteristics, negative events, individual emotions, and intention to drop out. Future research may need to verify these relationships by using quantitative methods. Such research may also help to address the limitations of using the AET theory, for example, doctoral students' intention to drop out might be associated with their socioeconomic status and the level of support from family and significant others.

## Data Availability

Publicly available datasets were analyzed in this study. This data can be found here: https://www.douban.com/group/657268/.
